# A Karnaugh map based approach towards systemic reviews and meta-analysis

**DOI:** 10.1186/s40064-016-2001-3

**Published:** 2016-03-25

**Authors:** Abdul Wahab Hassan, Ahmad Kamal Hassan

**Affiliations:** Saudi German Hospital, Jeddah, Saudi Arabia; Department of Electrical and Computer Engineering, King Abdulaziz University, Jeddah, Saudi Arabia

**Keywords:** Systemic reviews, Meta-analysis, Karnaugh map, Clinical coding, Reliability, Uncertainty

## Abstract

Studying meta-analysis and systemic reviews since long had helped us conclude numerous parallel or conflicting studies. Existing studies are presented in tabulated forms which contain appropriate information for specific cases yet it is difficult to visualize. On meta-analysis of data, this can lead to absorption and subsumption errors henceforth having undesirable potential of consecutive misunderstandings in social and operational methodologies. The purpose of this study is to investigate an alternate forum for meta-data presentation that relies on humans’ strong pictorial perception capability. Analysis of big-data is assumed to be a complex and daunting task often reserved on the computational powers of machines yet there exist mapping tools which can analyze such data in a hand-handled manner. Data analysis on such scale can benefit from the use of statistical tools like Karnaugh maps where all studies can be put together on a graph based mapping. Such a formulation can lead to more control in observing patterns of research community and analyzing further for uncertainty and reliability metrics. We present a methodological process of converting a well-established study in Health care to its equaling binary representation followed by furnishing values on to a Karnaugh Map. The data used for the studies presented herein is from Burns et al (J Publ Health 34(1):138–148, 2011) consisting of retrospectively collected data sets from various studies on clinical coding data accuracy. Using a customized filtration process, a total of 25 studies were selected for review with no, partial, or complete knowledge of six independent variables thus forming 64 independent cells on a Karnaugh map. The study concluded that this pictorial graphing as expected had helped in simplifying the overview of meta-analysis and systemic reviews.

## Background

Scientific research desires various methodologies to collect quality data, analyze it according to the required parameters and interpret the results of the data collected to formulate new tools, revise the existing guidelines, or simply verify current understandings. Studying trends in research across the regions or over a period of time in same geographic location by representing degree of variations on weighing various entities, which is mostly affected by evolution in knowledge and cultural trends besides other social factors, can give us a clue on preferences of the studied era or locality or both.

In a recent study (Davis et al. 2014) in SpringerPlus, analysis for systemic reviews and meta-analysis in social research was conducted. They developed an approach on dealing with multiple studies while developing meta-analysis and tried to answer basic four problems encountered in such scenarios. These problems include scoping and targeting of research questions appropriate for meta-analysis, selecting eligibility criteria where primary studies vary in design and choice of outcome measures, dealing with inconsistent reporting in primary studies, and identifying sources of heterogeneity with multiple confounded moderators. Their study however did not provide statistical tools to simplify the data collected from various studies, a visualization of multiple parameters used in different studies can potentially indicate the recent research trends.

The purpose of this study is to present the utility of Karnaugh map as a tool (Rushdi 1987; Miller et al. 2000; Holder 2005; Zhang 2009) that can pedagogically represent sparsely available statistic information. The rationale behind utilizing an engineering based mapping tools towards healthcare and specifically clinical coding lies in the fact that it would be a case study of humans’ pictorial perception and pattern recognition which can avoid complex computations and thus it provides a simplistic model that can easily be hand-checked. This presentation at the same time can also show the types of variables used in various studies, their levels of uncertainties (using Boolean function for dichotomous variables), the areas least studied or highly studied, and patterns of variable usage. To the best of author’s knowledge, currently no pictorial methodology was in place to judge the quality of studies included in a systemic review or a meta-analysis from clinical coding perspective. As an added value, availability of such tools can help us to compare and conclude graphically the quality of research conducted in different studies on a subject having common parameters. This can provide guidelines about specific variables in meta-analysis that can be prioritized in studies and also presents a clearer depiction of how the data varied among cases, thus helping in understanding the trends in scientific research. Other benefits of this tool are to identify which areas were minimally studied and which areas received greater attention.

The rest of the paper is organized as follows. “Overview of clinical coding” section deals with the overview of clinical coding and surveys most significant systemic reviews and meta-analysis. “A Karnaugh map based approach” section presents the Karnaugh-map based approach towards understanding the research directions while Abstraction, simulations in section is concerned with abstraction models and numeric simulation which is followed by, Discussions, Conclusions, Acknowledgements, Authors’ contributions and References.

## Overview of clinical coding

Clinical coding is a tool to indicate a specific code to a disease or a procedure, it can be used universally and is interpreted precisely and accurately every time. First such methodology was introduced by Jacques Bartillon in 1893 long after Florence Nightingale made a proposal on systemic collection of hospital data. Clinical coding thus, has been around for many decades and covers a big time span (Slee 1978; Butts and Williams 1982; Cimino et al. 1989; World Health Organization 1992, 2004; Steliarova-Foucher et al. 2005; Clark et al. 2010; Berger et al. 2015). One of the many coding systems in place is International Statistical Classification of Diseases and related health problems commonly known as International Classification of Diseases (ICD). It had its first classification in the year 1900 and it kept revising almost every 10 years. Since 1948, it is under the jurisdiction of World Health Organization (WHO-UN). ICD-10 was introduced in 1990 but its implementation started around 1994 and it is currently in place in most of the continents. The codes are alpha numeric patterns starting with an alphabet which usually signifies category (system involved in most cases) of disease and numeric value which identifies the true nature and stage of disease and if possible its laterality. Although the original idea behind such coding was to collect the data regarding the cause of deaths, as before the era of coding, different nomenclature was used in various localities making it difficult to aggregate or analyze data. Later the coding system included not only the cause of deaths but also disease of various systems. Collected data is used at different levels from hospital management to state policy making. The data is also critical since it will guide future planning and resource allocation for different units working in various areas of medicine.

A landmark study (Campbell et al. 2001), presented a systemic review related to UK data and showed an overall accuracy of collected data at about 84 %. Following the footsteps of Campbell et al., more recently another study (Burns et al. 2011) compared the various studies related to the accuracy of clinical coding related to diagnosis at discharge and concentrated primarily on analyzing the published accuracy of the collected data-sets in Great Britain. They collected data from various databases and methodologically included 25 studies in their research. Filtration process was such that 681 studies were excluded on basis of review of title and abstract while 37 papers were excluded when full papers were reviewed. The overall accuracy of the data collected according to this systemic review was 83 % where procedure accuracy was about 84.2 % while primary diagnosis coding was found to be 80.3 %. Although the two systemic reviews were comparable, the later had the recent data and updated protocols.

The data compiled by Burns et al. (2011) was assessed qualitatively using 6 variables (A–F) which included:

- A: Random sampling,
- B: At-least 90 % data sampled was available for analysis,
- C: Trained coders were utilized,
- D: Inter and Intra-Coder reliability,
- E: Awareness of the codes at the time of discharge, and
- F: Definition of accuracy.

All the 25 studies were tabulated with their pertinent variables (A–F) and were augmented by the year of the study and the data sources for each case labeled hereafter for convenience as

- X = Registry and case note
- Y1 = Case note review
- Y2 = Case note review and local registry
- Y3 = Operation-note review
- Z = Discharge summary

Table 1 reproduced) from Burns et al. (2011) displays data on stand-alone case-by-case basis to suggest how the data was complied with the given 6 parameters (A–F). By analyzing this table, the most controlled data where the researcher has knowledge of all 6 variables is case no. 25 (Colville and Laing 2000). The opposite is true for the case no. 19 (Samy et al. 1994) in which there is only one variable with a definite answer while all of the remaining 5 have uncertainties involved. The study conducted by case no. 8 (Dixon et al. 1998) is second most variable-aware. A thing of note here is that researcher’s awareness of a particular metric may or may not necessarily measure the overall accuracy of a study, though most affirmative variable knowledge can be used to make amends to overall accuracy on strictly statistical terms.

**Table 1:** 25 Selected studies related to clinical coding accuracy augmented with six independent variables

Case no.	Author’s	Data source	Random sampling (A)	90 % sampled data available (B)	Trained coders (C)	Coder reliability (D)	Coder awareness of codes (E)	Definition of accuracy (F)
1	Sellar et al. (1990)	X	No	Yes	Unclear	No	Yes, aware	Unclear
2	Smith et al. (1991)	Y1	Yes	Yes	No	No	Unclear	Unclear
3	Yeoh and Davies (1993)	Y1	Yes	No	Unclear	Yes	No, unaware	Unclear
4	Panayiotou (1993)	Y1	Unclear	Yes	Yes	No	Yes, aware	Three digit
5	Cleary et al. (1994)	Y1	Unclear	Unclear	Yes	Yes	Unclear	Four digit
6	Drennan (1994)	Y1	Yes	Yes	Yes	No	No, unaware	Unclear
7	Gibson and Bridgman (1998)	Y1	Yes	No	Unclear	No	Unclear	Four digit
8	Dixon et al. (1998)	Y1	Yes	Yes	Yes	Yes	Unclear	Four digit
9	Kirkman et al. (2009)	Z	Yes	Unclear	Unclear	No	Unclear	Four digit
10	Reddy-Kolanu and Hogg (2009)	Y1	Yes	Yes	Yes	No	Unclear	Unclear
11	Nouraei et al. (2009)	Y1	Yes	Yes	Yes	Unclear	Unclear	Four digit
12	Mitra et al. (2009)	Y1	Yes	Unclear	Yes	No	Unclear	Four digit
13	Beckley et al. (2009)	Y1	Yes	Unclear	Yes	No	Unclear	Unclear
14	Audit Commission (2010)	Y1	Yes	Unclear	Yes	Unclear	Unclear	Four digit
15	Murchison et al. (1991)	Y1	No	Yes	Unclear	No	Unclear	Unclear
16	Park et al. (1992)	Y1	No	Yes	No	No	Unclear	Unclear
17	McGonigal et al. (1992)	Y1	No	Yes	No	No	Yes, aware	Four digit
18	Pears et al. (1992)	Y1	Unclear	No	Unclear	Unclear	No, unaware	Four digit
19	Samy et al. (1994)	Y1	Yes	Unclear	Unclear	Unclear	Unclear	Unclear
20	Dornan et al. (1995)	Y1	Yes	Yes	Yes	No	Yes, aware	Unclear
21	Harley and Jones (1996)	Y1	Yes	Yes	Yes	No	Unclear	Three digit
22	Davenport et al. (1996)	Y2	No	Yes	Unclear	No	Yes, aware	Unclear
23	Kohli and Knill-Jones (1992)	Y1	Yes	Yes	Unclear	No	Yes, aware	Four digit
24	Hasan et al. (1995)	Y1	Yes	Yes	Yes	No	Unclear	Four digit
25	Colville and Laing (2000)	Y3	Yes	Yes	No	No	No, unaware	Four digit

## A Karnaugh map based approach

**Fig. 1:**
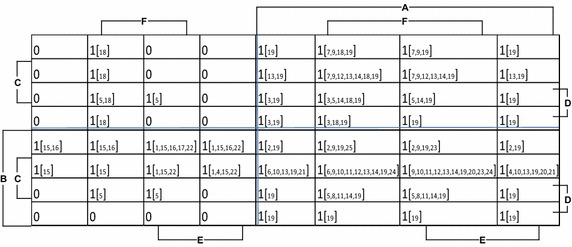
Karnaugh-map based representation of dichotomized data for Table 1

If one desires to know how many studies were conducted where a particular metric was always met or to check if the studies are overall in the right direction and are following the set guidelines in the best possible way, a simple truth-table like representation of Table 1 makes the analysis complex and hard to visualize. A possible way to deal with these questions is to initially convert the linguistic statement to Boolean values of 1 and 0. Affirmative statements such as Yes, Yes (aware), and Four digit of metrics (A–F) can be translated as 1 and negation statements of No, No (unaware), and Three digit can be expressed as a Boolean 0. A third value of metric related to uncertainty can be expressed as “X” or a *don’t care* in Boolean-logic terms. The analysis of such data with 6 variables for performance and each variable having 3 possible values can be solved using variable entered Karnaugh map (VEKM) and reader is suggested to (Holder 2005; Rushdi 1987; Rushdi and Amashah 2011) for a detailed description of VEKM. For the sake of simplicity, an alternate approach has been followed which lessens the control for analysis as compared to VEKM but yet provides a much broader and simplistic picture compared to Table 1. Presence of study is hereafter indicated by Boolean 1 which takes into account whether the author/authors of particular case knew about the parameters or not and the absence of study is taken as a crisp value of 0. By adopting this approach; linguistic terms of cases from Table 1 are translated to a Karnaugh-map liked structure presented in Fig. 1.

Formulation of Fig. 1 is based on four identical maps (Quadrants) stacked together thus forming a square like structure. The columns are furnished by variables A, E, and F while the rows are indicated by variables B, C, and D respectively. Each quadrant in the K-Map is organized based on a Gray code manner in such a way that if we hand pick any cell, the neighboring cell would differ in just one variable only. Taking the case of columns only, variables A, E, and F are appended from left to right in following order 000, 001, 011, 010, 100, 101, 111, and 110. Now assuming an absence of first variable A, we would have 00, 01, 11, and 10 presented twice as per the Gray code. A thing of note herein is that all cells are orthogonal with each other, thus allowing the possibility of arranging variables in multiple ways provided the orthogonality remains. Interested reader is suggested to two recent papers (Rushdi and Hassan 2015, 2016a) with extensive manipulation based on Karnaugh Maps.

From clinical coding perspective, each of the study can be translated as a Boolean dichotomized function which can be plotted on a Karnaugh map. It is essentially a graphical representation comprising of a two dimensional rectangular grid where each of the squares is representing the different combination of the variables or performance metrics in the present case. The Karnaugh map in Fig. 1 comprises of 2^*n*^ cells, where *n* represent the number of performance variables. In the present study, *n* = 6, resulting in a grid of 64 cells. It can be seen that various cells have no study whatsoever and thus have an assigned value of “0”, it is to be noted here that this is a crisp value indicating independence from knowledge of parameters for all cases of Table 1. If a cell representing a set of variables has been studied once or more, again independent from the knowledge of parameters involved, it will be valued one “1_*k*_”, where *k* represents the case number of study from Table 1 which has met the cell criteria. From the Karnaugh map presented in Fig. 1, we can easily take any study and see how many variables a study is addressing with or without certainty, e.g., the cell in the top left corner represents none of the required variables are met while the cell in the bottom right corner require an affirmative value of three variables namely A, B, and D. The cell with arrow requires all the 6 variables to be met in affirmation. So, this is our most valuable cell and the immediate neighboring cells should be second most important compared to other cells. If we look into the marked cell, there are 5 studies (5, 8, 11, 14, and 19). If we look into the surrounding cells, we notice that a large number of studies fall around this cell especially on the immediate upper cell. Resultantly, this can give us a better understanding of research behavior and the preferred importance of metrics which are being studied more and this representation also shows where there has been for any reason minimal research. The highest number of studies (10) is found in the cell above the marked cell.

Figure 1, if used in collaboration with Table 1, will show the quality of studies conducted with specific parameters of any cell, e.g. study no 8 and 19 are from the most valued cell. From Table 1, we know that the study with serial number 8 (Dixon et al. 1998) has 5 definite answers and only one unclear answer, while no. 19 (Samy et al. 1994) from the same box has only one definite response and 5 unclear values. So, the former has a data which may be relevant and accurate to a greater degree while the later has although relevant data, it is extremely unreliable.

**Fig. 2:**
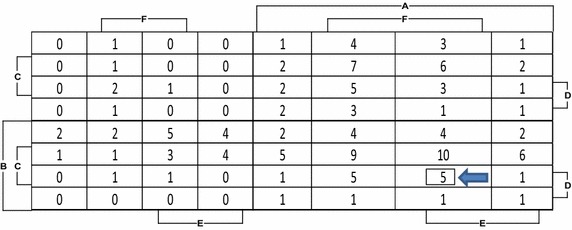
A weighted Karnaugh-map like representation representing the significance of cells in research methodology

To make things simpler for systemic reviews or meta-analysis and to visualize the areas of concentration of studies, we can give numerical values to each cell based on the number of studies conducted in it. From the map above containing serial number of the studies in each cell, the summarized map presented in Fig. 2 has been extracted by numeric mapping method and it can be observed that 5 studies are present in the marked cell and 34 representations are found in the surrounding cells. A very obvious finding is that the cells in the row below although should also be heavily populated have only one study which is case no. 19. The rationale behind this unusual research behavior can be explained if we look into these cells and see which variable is missing. These cells had studies with negative response to variable “C” which was utilization of the trained coder. Although, all variables have their importance but the unavailability of a trained coder can affect the study in the worst possible ways as the personnel who had received informal experience or training and are not well trained in the coding methodology are likely to be unaware of the coding standards in detail which can lead to wrong coding in most scenarios.

## Abstraction, simulations

Overall research methodology can be expressed in terms of a closed form expression by resorting to Karnaugh map presented in Fig. 3. We have made 5 disjoint loops thus giving as a function of Research Methodology *R*_*m*_ over the investigated period as

1 $$R_{m} \left\{ 1 \right\} = A \vee \bar{A} B\bar{D} \vee \bar{A}\bar{B}\bar{E} F \vee \bar{A} B C D F \vee \bar{A}\bar{B} C D E F$$

**Fig. 3:**
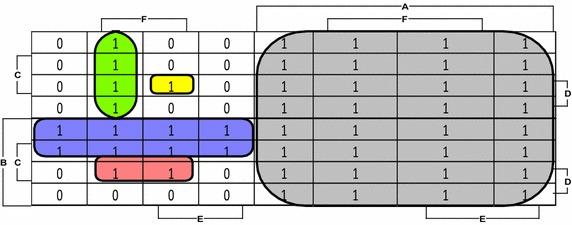
A Karnaugh-map representation with disjoint loops for indicating research methodology of Table 1

It can be noted that the Karnaugh map representation of Fig. 3 can further provide a more minimal expression by relaxing the condition of disjoint loops. One such expression can be

2 $$R_{m}^{{\prime }} \left\{ 1 \right\} = A \vee B\bar{D} \vee \bar{A}\bar{B}\bar{E} F \vee \bar{A} B C F \vee \bar{A}\bar{B} C D F$$

The expression (2) is certainly more minimal expression than (1) and is more efficient in terms of computational complexity, but it is not disjoint, the downside is that it cannot be directly converted to a probability ready expression defined as (Rushdi and Hassan 2015) “An expression in the switching (Boolean) domain, in which logically multiplied (ANDed) entities are statistically independent and logically added (ORed) entities are disjoint. Such an expression can be directly transformed, on a one-to-one basis, to the algebraic or probability domain by replacing switching (Boolean) indicators by their statistical expectations, and also replacing logical multiplication and addition (ANDing and ORing) by their arithmetic counterparts”.

Since the expression (1) is in disjoint form, hence it is known as Probability Ready Expression (PRE), the conversion of such PRE is straightforward now.

3 $$p_{{R_{m}}}\left\{ 1 \right\} = p_{A} + q_{A} p_{B} q_{D} + q_{A} q_{B} q_{E} p_{F} + q_{A} p_{B} p_{C} p_{D} p_{F} + q_{A} q_{B} p_{C} p_{D} p_{E} p_{F}$$

Expression (3) presents a probability function for availability of research thus giving the performance metric values for a whole course of universe. Further to quantify the distribution of $p_{{R_{m} }} \left\{ 1 \right\}$ in expression (3) with each variable having an embedded uncertainty involved, we resort to the method of uncertainties in distribution (Rushdi 1985; Rushdi and Ba-Rukab 2005a, b; Forbes et al. 2011; Rushdi and Hassan 2016b). Assuming that each variable (A–F) is identically and log-normally distributed having a mean value of 0.5 and variance of 0.005, using Monte Carlo simulation for sample size 100,000 and using commercial MATLAB^®^ software package, we will have the resultant moments for $p_{{R_{m} }} \left\{ 1 \right\}$ of expression (3). Numerical results for the first two moments mean and variance are $\mu_{1} = 0.7344$ and *μ*_2_ = 0.0020 respectively. Further by utilizing the dimensionless coefficients of variation ($\rho = \mu_{2}^{1/2} / \mu_{1}$ = 0.0609), skewness ($\gamma_{1} = \mu_{3} /\mu_{2}^{{\frac{3}{2}}} = 0.0740)$ and excess (kurtosis) ($\gamma_{2} = \frac{{\mu_{4} }}{{\mu_{2}^{2} }} - 3 = 3.0655)$ we are able to calculate third and fourth central moments as $\mu_{3} = 6.6360{\text{e}} - 06$ and $\mu_{4} = 2.4339{\text{e}} - 05$ respectively. Figure 4 presents a histogram for the expression (3) with uncertainty involved in parameters.

**Fig. 4:**
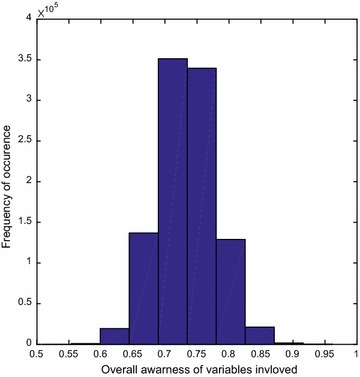
A histogram representing the effect of uncertainties of individual metrics translated onto overall uncertainty in research methodology

## Discussions

Whenever a systemic review or a meta-analysis is conducted, generally a table is organized from the results of various studies where each study is indexed along with linguistic or numeric range of values of certain specific parameters. If we need to observe each study independently, it can be done in the conventional tabulated pattern i.e. by focusing on one study at a time. Further, a comparison can be made with second, third, or few studies at best in such presentation of data. On the other hand, if we want to review multiple studies together, uni-lateral tables desire serious pictorial ingenuity in extracting parallels and contrasts. In fact, it is often cumbersome and unrealistic to count the utilization of each variable for each study on meta-scale, a thing often reserved on the computational power of the machines.

To this end, we presented the utility of a Karnaugh map based approach for organizing meta-data based on specific number of variables which has potential to not only simplify the collected data into pictorial presentation but also show us the areas of maximal or minimal research activity. One may also look into groups of plotted studies to find specific trends. There are certainly other tools of mapping such as Venn Diagram and Time-distance diagram but these are beyond the control of human perception specially for big data analysis. Another important feature of utilizing a Karnaugh map based approach is that the K-map works on prime implicants that can easily be extracted using custom based software such as MATLAB^®^ and TOSMANA and have a plethora of algorithms such as Quine-McCluskey and ESPRESSO.

## Conclusions

The availability of mapping tools that are simplistic in nature, computationally efficient, and well established in engineering disciplines are pedagogically presented herein for the systemic reviews and meta-analysis. A landmark work on clinical coding with six independent variables involved; conventionally displayed on a uni-lateral tabulation are systematically transformed herein on to a Karnaugh-map. This tool serves as an indicator function in understanding the variable utilization, credibility of collected data, and quality of studies included in Clinical coding. K-Maps can potentially address systemic reviews and meta-analysis with conflicting results by comparing the number of credible studies included in each analysis and also in directing us towards the areas least and most studied. It also draws a number of remarks on observing ongoing shifts in research orientations, current trends, and past practices. We thus conclude that a Karnaugh Map is a useful statistical tool which can be recasted in Social and Medical sciences to simplify the analysis of collected data.
